# Long-term safety of budesonide/formoterol for the treatment of elderly patients with bronchial asthma

**DOI:** 10.3892/etm.2014.1515

**Published:** 2014-01-30

**Authors:** KATSUNORI KAGOHASHI, HIROAKI SATOH, GEN OHARA, KUNIHIKO MIYAZAKI, MIO KAWAGUCHI, KOICHI KURISHIMA, NOBUYUKI HIZAWA

**Affiliations:** 1 Division of Respiratory Medicine, Mito Medical Center, University of Tsukuba, Mito, Ibaraki 310-0015, Japan; 2 Division of Respiratory Medicine, Faculty of Medicine, University of Tsukuba, Tsukuba, Ibaraki 305-8575, Japan

**Keywords:** bronchial asthma, budesonide/formoterol, inhalation, elderly, pulse rate, serum potassium

## Abstract

The long-term safety of budesonide/formoterol (BUD/FM) inhalation has not been fully evaluated, particularly in elderly patients with bronchial asthma. To evaluate the 12-month safety of BUD/FM inhalation for elderly asthmatic patients, the changes in serum potassium levels and pulse rate were examined. A retrospective chart review was conducted of consecutive patients who were treated with BUD/FM inhalation (two inhalations of 160/4.5 mg, twice daily; Symbicort Turbuhaler, AstraZeneca) at a hospital between February 2010 and January 2012. A total of 350 patients were treated with BUD/FM inhalation during the study period and were followed up over 12 months. The mean age of the patients was 60 years, and 19.4% and 21.4% of the patients were aged 65–74 years and ≥75 years, respectively. One hundred and fourteen (32.6%) of the 350 patients continued the inhalation therapy for >12 months. Compared with the pretreatment data, reductions in serum potassium levels at 1, 6 and 12 months were not observed, even in the patients aged 65–74 and ≥75 years. There was also no increase in the pulse rate at 1, 6 and 12 months, even in the patients aged 65–74 and ≥75 years. The usual dosage of BUD/FM showed no adverse effects on the serum potassium levels and pulse rate in the adults, including the elderly with persistent asthma.

## Introduction

The use of an inhaled corticosteroid (ICS) and long-acting β2-agonist (LABA) combination inhaler is recommended by the Global Initiative for Asthma and most other asthma treatment guidelines as the first choice to control chronic asthma for patients in whom control with ICS monotherapy is difficult (1–4). Budesonide/formoterol (BUD/FM) inhalation aerosol is an ICS and LABA combination, which is administered twice daily via one hydrofluoroalkane-pressurized metered-dose inhaler and has been approved for use in many countries for the long-term maintenance treatment of persistent asthma (2–4). With regard to safety, while the short-term safety in ICS (5–7) and ICS/LABA (8–10) has been evaluated, the long-term safety has yet to be fully investigated (11,12), particularly in elderly patients with bronchial asthma. In the present retrospective study, an evaluation of the 1-, 6- and 12-month safety of BUD/FM inhalation for elderly asthmatic patients was performed, and the changes in serum potassium levels and pulse rate were observed.

## Patients and methods

### Patients

Clinicopathological data for all the patients with bronchial asthma were obtained by retrospective review from the database at the University of Tsukuba, Mito Medical Center, Mito Kyodo General Hospital (Mito, Japan). The consecutive patients who were diagnosed with bronchial asthma and treated with BUD/FM inhalation aerosol (two inhalations of 160/4.5 mg, twice daily; Symbicort Turbuhaler; AstraZeneca, Osaka, Japan) between February 2010 and January 2012 at the hospital, were entered in this study.

In order to evaluate the 1-year safety of BUD/FM inhalation, a medical chart review of the patients up to January 2013 was performed. Demographic data, including age, gender and comorbid diseases were retrieved from the patient medical records. This retrospective study conformed to the Ethical Guidelines for Clinical Studies issued by the Ministry of Health, Labor and Welfare of Japan.

### Study population

The patient population was divided into three age groups: The <65 years group, the 65–74 years group and the ≥75 years group. The demographic data and safety of BUD/FM inhalation therapy were compared among the three age groups. Adverse effects were counted as they occurred during the study period. Blood samples were obtained from the patients in order to measure the serum potassium levels, and pulse rates were measured at pretreatment and 1, 6 and 12 months following the initiation of the BUD/FM inhalation therapy.

### Statistical analysis

The serum potassium levels and pulse rate prior to treatment and at 1, 6 and 12 months after treatment were compared using Wilcoxon rank sum test. To compare the different groups of patients, the chi-square test was also used. All statistical analysis were performed using SPSS software, version 10.1 for Windows (SPSS Inc., Chicago, IL, USA) and P<0.05 was considered to indicate a statistically significant difference.

## Results

### Patient characteristics

Table I shows patient characteristics. A total of 350 patients with bronchial asthma were treated with BUD/FM inhalation during the study period. There were 189 males and the median age was 60 years (range: 17–93 years). In total, 207 (59.2%) patients were aged <65 years, 68 (19.4%) patients were 65–74 years old and 75 (21.4%) patients were ≥75 years old (Table I). Among the 350 patients, 160 (45.7%) patients had comorbid diseases, including cardiovascular diseases in 74 patients (54 patients had hypertension, 12 had chronic heart failure, six had arrhythmia and five had ischemic heart disease). Fifty-six patients (74.7%) aged ≥75 years had one or more comorbid diseases, and 64.7% and 24.1% of patients aged 65–74 years and <65 years had one or more comorbid diseases, respectively. There was a significant difference in the incidence of one or more comorbid diseases between patients aged ≥65 years and those aged <65 years (P=0.001; chi-square test). However, there was no difference in the incidence of comorbid diseases between patients aged ≥65–74 years and those aged <75 years (P=0.603; chi-square test).

### Treatment of bronchial asthma

An effective control of bronchial asthma was obtained, so BUD/FM inhalation therapy in 141 (40.3%) of the 350 patients was terminated or changed to ICS inhalation within 3 months. Similarly, in 95 (27.1%) patients, the inhalation therapy was terminated within 3–12 months. Therefore, the inhalation therapy was continued for >12 months in 114 (32.6%) patients.

### Safety of BUD/FM inhalation

#### Adverse events

Four (1.1%) patients exhibited hoarseness, and three of them were aged ≥75 years. Two (0.6%) patients developed a tremor and both were <75 years of age. Two (0.6%) patients had oral candidiasis and arrhythmia, and each of them was ≥75 years of age. All adverse events were transient and disappeared shortly after the termination of the inhalation therapy.

#### Serum potassium

**Figure 1:**
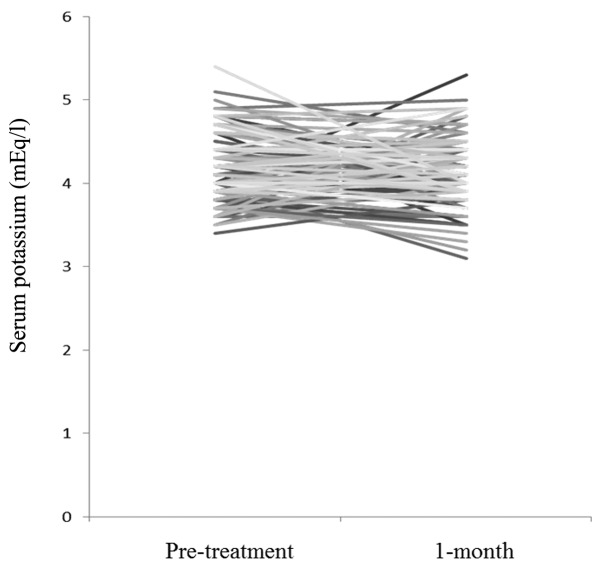
Serum potassium levels prior to and 1 month after the initiation of BUD/FM inhalation. No statistically significant difference was identified (P=0.567). BUD/FM, budesonide/formoterol.

Fig. 1 shows the changes in serum potassium levels between pretreatment and 1 month after the initiation of BUD/FM therapy. There was no statistical difference between them (P=0.567). As shown in Table II, serum potassium levels at 6 and 12 months were not different from the levels before treatment (P=0.941 and P=0.822, respectively). In the 65–74 years group and the ≥75 years group, serum potassium levels at 1, 6 and 12 months was not different from the levels before treatment. The changes in serum potassium levels in patients with cardiovascular diseases, and those ≥75 years with comorbid diseases were also examined. There was no statistical differences between serum potassium levels at these intervals (Table II).

#### Pulse rate

**Figure 2:**
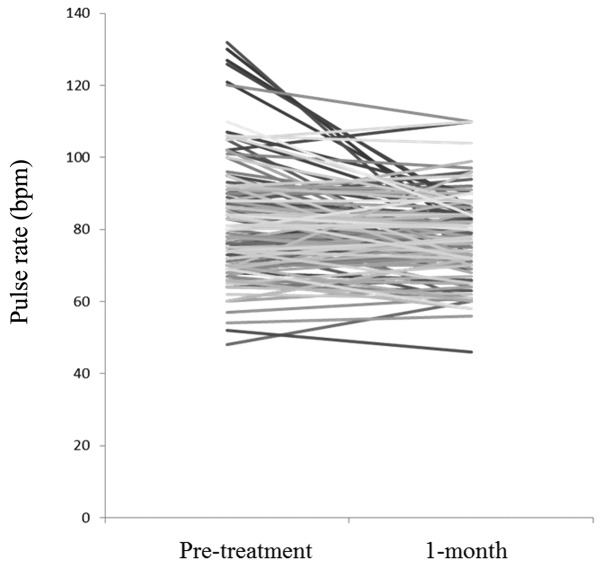
Pulse rate prior to and 1 month after the initiation of BUD/FM inhalation. The pulse rate 1 month after the BUD/FM inhalation was significantly lower than that after treatment (P=0.001). BUD/FM, budesonide/formoterol.

Fig. 2 shows changes in pulse rate between pretreatment and 1 month after the initiation of BUD/FM therapy. The pulse rate 1 month after the initiation of BUD/FM therapy was significantly lower than the pulse rate prior to treatment (P=0.001). In the ≥75 years group, the pulse rate was also decreased at the 6-month interval (P=0.046); these results may be due to the achievement of control of bronchial asthma. However, there was no statistically significant difference in pulse rate between any other age groups or intervals (Table III). The change in pulse rate in asthmatic patients with cardiovascular diseases and those ≥75 years with comorbid diseases were also examined, and no statistical differences between any age groups and any intervals were identified (Table III).

## Discussion

BUD, a potent and safe ICS with a high affinity for glucocorticoid receptors, is approved in many countries for the treatment of asthma (2–4). The addition of inhaled LABA provides a more effective means of improving lung function and asthma control than increasing the dose of ICS in patients whose asthma is not adequately controlled. FM is unique amongst LABAs as it has a fast onset of action and a long duration of effect (2,3). BUD and FM, as individual components and in combination, possess well-defined efficacy as well as pharmacological and safety profiles when administered through a single metered-dose inhaler (4,8–10). In the present study, an evaluation of safety over 12 months of the usual dose of BUD/FM was performed, and the serum potassium levels and pulse rate were examined in elderly patients with bronchial asthma.

There has been increasing interest in the treatment of elderly patients with bronchial asthma (13). However, clinical information regarding asthmatic patients aged ≥75 years has been scarcely available as such patients are not usually included in clinical trials and retrospective care analysis. Therefore, there is scientific uncertainty regarding the risks and benefits of treatment with ICS/LABA in asthmatic patients aged ≥75 years. To assess the short-term efficacy and safety of the treatment in the elderly, we previously reviewed our clinical data from consecutive patients with bronchial asthma treated with BUD/FM (14). Even with the existence of comorbid diseases in the elderly, BUD/FM was effective with no high incidences of adverse events, such as hoarseness, tremor, arrhythmia and oral candidiasis (14). The incidences observed were similar to those reported in previous studies in young adults and middle-aged patients (8–10).

Previous studies have investigated the 1-year safety of the usual dosage of FM (5–7) and BUD/FM inhalation for asthma patients (8–10). In these studies, the mean age of the patients evaluated was ~30–40 years (8–10). Rosenhall *et al* evaluated the 1-year safety of BUD/FM (8), and Maspero *et al* examined mometasone furoate/FM (9), and they concluded that both treatments were safe and well tolerated in patients with persistent asthma (8,9). However, in these studies, there was no investigation into the changes in serum potassium levels and pulse rate. With regard to the evaluation of these parameters, Hinkle *et al* studied heart rate in pediatric subjects with stable asthma (15), and Malolepszy *et al* examined serum potassium levels and heart rate in high-dose FM in patients with acute bronchial obstruction (16). Recently, Saito and Hasunuma studied the short-term safety of high dose BUD/FM in asthmatic patients, whose mean age was 44.3 years (11). In their study, >10% of patients showed palpitation, tachycardia and decreased serum potassium levels in a 2.5-fold higher dose of BUD/FM than the usual dose of therapy (11). In the present study, the median age of the patients was 60 years, and 40.8% and 21.4% of them were ≥65 and ≥75 years of age, respectively. Aging is associated with a high prevalence of comorbid diseases (13). Elderly patients are predisposed to comorbid diseases, such as diabetes, cardiovascular and cerebrovascular diseases. In the present study, the ≥65 years group had a higher proportion of patients with comorbid diseases than those <65 years of age. However, there were neither significant reductions in serum potassium levels nor changes in pulse rate in the three age groups, including those with comorbid diseases.

The results of the present study revealed novel findings; however, there were a number of limitations in this study. This was a small-sized retrospective study in a single institution. Serum potassium levels and pulse rate were not evaluated in every patient and a quality of life analysis was not performed in this study. Therefore, we consider that conclusive outcomes were not derived from this study. However, the methodology used, which was based on an audit of the information from clinical practice documented in the patient records, may provide some clinical information that was not available from clinical trials. Additionally, reporting the treatment experiences in patients with bronchial asthma, including those of elderly patients with some comorbid diseases, may be of clinical significance.

In conclusion, the usual dosage of BUD/FM (two inhalations of 160/4.5 mg, twice daily) showed no adverse effects on the serum potassium levels and pulse rate in Japanese adults, including the elderly with persistent asthma.

## Figures and Tables

**Table I:** Characteristics of 350 patients with bronchial asthma.

Variable	Data
Age (years)	60 (17–93)^a^
≤65	207 (59.2%)
65–74	68 (19.4%)
≥75	75 (21.4%)
Gender	
Male	189
Female	161
Comorbid diseases	
Present	160 (45.7%)
Cardiovascular diseases	74
Hypertension	54
Chronic heart failure	12
Arrhythmia	6
Ischemic heart disease	5
Others	4
Respiratory diseases	43
COPD	26
Pneumonia	5
Others	8
Metabolic diseases	43
Diabetes	29
Thyroid disease	5
Others	9
Renal and urologic diseases	14
Autoimmune diseases	9
Psychiatric diseases	8
Malignant diseases	7
Other diseases	14
Other controllers	
Leukotriene antagonists	41
Xanthines	19
Oral steroids	2

COPD, chronic obstructive pulmonary disease.

a median with range in parentheses.

**Table II:** Change in serum potassium levels prior to and following BUD/FM inhalation.

Patients	Pretreatment K level (median, range; mEq/l)	Post-treatment K level (median, range; mEq/l)	P-value
All patients			
1-month interval	4.1, 3.4–5.4	4.2, 3.1–5.3	0.567
6-month interval	4.1, 3.4–5.0	4.1, 3.5–5.1	0.941
12-month interval	4.2, 3.4–5.4	4.2, 3.5–5.2	0.822
Patients aged ≥65 years			
1-month interval	4.2, 3.5–5.4	4.2, 3.6–4.9	0.286
6-month interval	4.2, 3.5–5.0	4.1, 3.6–5.1	0.457
12-month interval	4.2, 3.4–5.4	4.3, 3.5–5.2	0.989
Patients aged ≥75 years			
1-month interval	4.2, 3.6–5.4	4.2, 3.6–4.9	0.691
6-month interval	4.1, 3.6–5.0	4.1, 3.6–4.8	0.909
12-month interval	4.1, 3.4–5.4	4.3, 3.5–5.0	0.472
Patients with cardiovascular diseases			
1-month interval	4.1, 3.4–4.9	4.3, 3.2–5.0	0.680
6-month interval	4.1, 3.5–5.0	4.3, 3.6–5.1	0.345
12-month interval	4.1, 3.0–5.0	4.2, 3.6–5.0	0.731
Patients ≥65 years with cardiovascular diseases			
1-month interval	4.2, 3.7–4.9	4.3, 3.5–4.9	0.967
6-month interval	4.3, 3.7–5.0	4.1, 3.6–5.1	0.795
12-month interval	4.3, 3.7–5.0	4.3, 3.7–5.0	0.948

BUD/FM, budesonide/formoterol; K, potassium.

**Table III:** Change in pulse rates prior to and following BUD/FM inhalation.

Patients	Pretreatment pulse rare (median, range; bpm)	Post-treatment pulse rate (median, range; bpm)	P-value
All patients			
1-month interval	80, 48–132	77, 46–110	0.001
6-month interval	81, 48–120	80, 52–120	0.081
12-month interval	80, 48–120	77, 50–109	0.050
Patients aged ≥65 years			
1-month interval	78, 54–110	77, 56–110	0.210
6-month interval	81, 54–106	76, 56–105	0.163
12-month interval	80, 54–106	76, 56–109	0.288
Patients aged ≥75 years			
1-month interval	81, 60–110	78, 58–110	0.093
6-month interval	81, 60–106	76, 56–105	0.046
12-month interval	81, 60–106	77, 56–109	0.128
Patients with cardiovascular diseases			
1-month interval	80, 52–120	79, 46–110	0.140
6-month interval	80, 54–120	80, 56–120	0.915
12-month interval	78, 60–120	77, 60–92	0.762
Patients aged ≥65 years with cardiovascular diseases			
1-month interval	81, 54–102	80, 56–97	0.325
6-month interval	81, 54–102	80, 56–98	0.726
12-month interval	80, 54–102	76, 58–92	0.807
